# AI-Based 3D-Segmentation Quantifies Sarcopenia in Multiple Myeloma Patients

**DOI:** 10.3390/diagnostics15192466

**Published:** 2025-09-26

**Authors:** Thuy-Duong Do, Tobias Nonnenmacher, Marieke Burghardt, Stefanie Zschaebitz, Marina Hajiyianni, Elias Karl Mai, Marc-Steffen Raab, Carsten Müller-Tidow, Hans-Ulrich Kauczor, Hartmut Goldschmidt, Ulrike Dapunt

**Affiliations:** 1 Clinic of Diagnostic and Interventional Radiology (DIR), Heidelberg University Hospital, 69120 Heidelberg, Germany; 2 Department of Nuclear Medicine, University Hospital Heidelberg, 69120 Heidelberg, Germany; 3 German-Speaking Myeloma Multicenter Group (GMMG), Heidelberg University Hospital, 69120 Heidelberg, Germany; 4 Heidelberg Myeloma Center, Department of Internal Medicine V, Hematology, Oncology and Rheumatology, Medical Faculty Heidelberg, Heidelberg University Hospital, 69120 Heidelberg, Germany; 5 National Center for Tumor Diseases (NCT), Department of Medical Oncology, Heidelberg University Hospital, 69120 Heidelberg, Germany

**Keywords:** tomography, X-ray computed, multiple myeloma, sarcopenia, induction chemotherapy, intelligent systems

## Abstract

**Background:** Sarcopenia is characterized by a loss of muscle mass and strength, resulting in functional limitations and an increased risk of falls, injuries and fractures. The aim of this study was to obtain detailed information on skeletal muscle changes in patients with multiple myeloma (MM) during treatment. **Methods:** A total of 51 patients diagnosed with MM who had undergone whole-body low-dose computed tomography acquisition prior to induction therapy (T1) and post autologous stem cell transplantation (T2) were examined retrospectively. Total volume (TV), muscle volume (MV) and intramuscular adipose tissue volume (IMAT) of the autochthonous back muscles, the iliopsoas muscle and the gluteal muscles were evaluated on the basis of the resulting masks of the BOA tool with the fully automated combination of TotalSegmentator and a body composition analysis. An in-house trained artificial intelligence network was used to obtain a fully automated three-dimensional segmentation assessment. **Results:** Patients’ median age was 58 years (IQR 52–66), 38 were male and follow-up CT-scans were performed after a mean of 11.8 months (SD ± 3). Changes in MV and IMAT correlated significantly with Body-Mass-Index (BMI) (r = 0.7, *p* < 0.0001). Patients (*n* = 28) with a decrease in BMI (mean −2.2 kg/m^2^) during therapy lost MV (T1: 3419 cm^3^, IQR 3176–4000 cm^3^ vs. T2: 3226 cm^3^, IQR 3014–3662 cm^3^, *p* < 0.0001) whereas patients (*n* = 20) with an increased BMI (mean +1.4 kg/m^2^) showed an increase in IMAT (T1: 122 cm^3^, IQR 96.8–202.8 cm^3^ vs. T2: 145.5 cm^3^, IQR 115–248 cm^3^, *p* = 0.0002). Loss of MV varied between different muscle groups and was most prominent in the iliopsoas muscle (−9.8%) > gluteus maximus (−9.1%) > gluteus medius (−5.8%) > autochthonous back muscles (−4.3%) > gluteus minimus (−1.5%). Increase in IMAT in patients who gained weight was similar between muscle groups. **Conclusions:** The artificial intelligence-based three-dimensional segmentation process is a reliable and time-saving method to acquire in-depth information on sarcopenia in MM patients. Loss of MV and increase in IMAT were reliably detectable and associated with changes in BMI. Loss of MV was highest in muscles with more type 2 muscle fibers (fast-twitch, high energy) whereas muscles with predominantly type 1 fibers (slow-twitch, postural control) were less affected. This study provides valuable insight into muscle changes of MM patients during treatment, which might aid in tailoring exercise interventions more precisely to patients’ needs.

## 1. Introduction

Sarcopenia manifests as a progressive decline in both muscle mass and strength as individuals age, accompanied by functional impairment, heightened susceptibility to falls and elevated fracture risk [1]. These alterations may also arise as secondary manifestations of malignancy, precipitated by an accompanying systemic inflammatory response [2]. Moreover, sarcopenia has been described to correlate with diminished overall survival rates, heightened chemotherapy-related toxicity and accelerated disease advancement among cancer patients [3]. Several studies investigated the effect of sarcopenia on clinical outcomes of patients with hematologic malignancies [4,5,6,7]. For example, Williams et al. showed that sarcopenia, defined as ≤80% high-density muscle, was found in 51% of multiple myeloma (MM) patients post-transplant and was associated with early cardiovascular complications [8]. No effect on overall survival was observed which is in line with another study by Abdallah et al. [9].

Multiple myeloma (MM) is a disease characterized by the monoclonal expansion of plasma cells in the bone marrow which might result in numerous osteolytic bone lesions and hence instability of the skeletal system [10,11,12]. Therefore, loss of muscle mass and strength is particularly critical for MM patients, because functional impairment and an increased risk of falls might result in detrimental injuries. In order to minimize risk of injuries, assessment and subsequent treatment of sarcopenia are highly relevant in MM.

Most studies have analyzed a single cross-sectional computed tomography (CT) image at the third lumbar vertebra in order to estimate whole-body muscle volume. In order to acquire more detailed information on muscle changes in MM patients during treatment, we were interested in measuring entire muscle groups. MM patients are usually examined by whole-body CT at initial diagnosis and after autologous stem cell transplantation, which offer an ideal opportunity to thoroughly investigate muscle changes during the treatment course. Our aim was to acquire in-depth information on muscle changes in MM patients during treatment.

Consequently, a three-dimensional comprehensive methodology was established for the assessment of muscle volumes in patients afflicted with MM. Manual segmentation requires trained radiologists, entails substantial time investment, and thus incurs considerable financial expenses and particularly poses challenges when applied to extensive image datasets, such as whole-body imaging or large cohort studies. Recent investigations have integrated automated segmentation methodologies, leveraging atlas-based segmentation [13,14], statistical shape modeling [15,16] or machine learning techniques [17,18], employing both two-dimensional and three-dimensional segmentation algorithms. Numerous deep learning (DL) models have emerged, primarily emphasizing the enhancement of training evaluation metrics, whose broader applicability might be constrained by specific data assumptions and frequently unrecorded configurations and may lack anatomical precision. For this study, we therefore chose to use the nnU-Net as it facilitates cross-task generalization and is focused on the evolution towards a data-centric artificial intelligence in radiological applications [19].

## 2. Material and Methods

### 2.1. Patients

This retrospective study was performed according to the Declaration of Helsinki and was approved by the local ethics committee (No. S-096/2017, S-195/2025), which waived the requirement for informed consent. Data of MM patients (*n* = 51) who received low-dose whole-body CT-scans at initial diagnosis (T1) and after high-dose chemotherapy/autologous stem cell transplantation (T2) were analyzed retrospectively. CT-scans were conducted at the multiple myeloma outpatient department between March 2018 and June 2023.

### 2.2. Clinical Data Selection and Study Design

For the study enrollment, the hospital’s information systems (I.S.-H.*med., SAP and Centricity RIS-i, GE Healthcare) were screened for eligible patients to ensure inclusion criteria were met. Patients diagnosed with MM who had undergone both initial and follow-up whole-body low-dose CT acquisitions prior to induction therapy and post autologous stem cell transplantation were enrolled in the study. Exclusion criteria were missing imaging data, contrast media enhanced CT acquisitions and missing clinical data. Patients’ clinical data at initial diagnosis and at a follow-up visit 2–3 months after autologous stem cell transplantation were evaluated retrospectively.

### 2.3. Computed Tomography Acquisition

Non-enhanced CT acquisitions were conducted in helical mode, spanning from the vertex of the skull to the knees, utilizing a dual-layer detector technique (IQon Spectral CT, Philips, Amsterdam, The Netherlands). The acquisition parameters were as follows: tube potential set at 120 kV_p_, dose right index of 15 (employing automated attenuation-based dose modulation), average tube current-time product of 93 mAs, volumetric computed tomography dose index (CTDI_vol_) measuring 8.4 mGy, pitch set at 1.0, gantry rotation time of 0.75 s and collimation dimensions of 64 × 0.625 mm.

### 2.4. Segmentation

**Figure 1:**
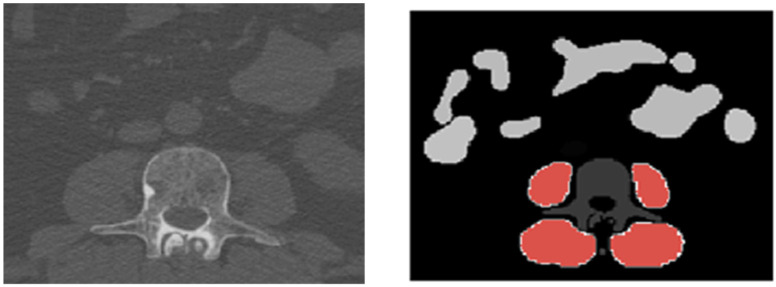
Example of muscle segmentation. (**left**) Axial view of whole-body low-dose CT without contrast agent at the level of the first lumbar vertebra. (**right**) AI-based segmentation of autochthone muscle and psoas muscle (red) with a total volume of 1149 cm^3^: vertebra in dark grey and bowels in light grey.

Volumetric delineation and quantification of muscle tissue rely on slice-by-slice semantic segmentation of muscle compartments. To achieve comprehensive segmentation, the open-source body and organ analysis (BOA) platform was employed [20]. BOA combines two algorithms: TotalSegmentator [21] and body composition analysis (BCA) [22]. BCA underwent training utilizing the nnU-Net framework, chosen for its robustness in accommodating diverse image dimensions and delivering adequate automated segmentation performance [19]. A manual validation process was conducted to verify the accuracy of all predicted segmentation masks in conjunction with the imaging data, ensuring the exclusion of grossly inaccurate segmentation predictions from feature extraction and preventing the use of corrupted raw imaging data (Figure 1).

### 2.5. Statistical Analysis

Data were summarized by descriptive statistics and differences between groups were calculated by the Wilcoxon matched-pairs signed-rank test and the Mann–Whitney test, respectively, using Graph Pad Prism 10 software (Graph pad software, Boston, MA, USA). Spearman correlation was computed to evaluate an association between changes in Body-Mass-Index and changes in total volume. The significance level was determined as *p* < 0.05.

## 3. Results

Clinical data of patients included in this analysis (*n* = 51) are listed in Table 1. Patients‘ median age was 58 years (IQR 52–66) and 75% of patients were male, 25% female. All patients underwent induction therapy followed by single (*n* = 24) or tandem (*n* = 27) high-dose chemotherapy/autologous stem cell transplantation. All patients received glucocorticoids during treatment. A follow-up CT-scan at T2 was conducted after a mean of 11.8 months (SD ± 3).

Different muscle groups were analyzed by means of an artificial intelligence-based three-dimensional segmentation process. The parameters total volume (TV), muscle volume (MV) and intramuscular adipose tissue volume (IMAT) were quantified (all in cm^3^) in the autochthonous back muscles (ABM), iliopsoas muscle (IM), as well as the gluteus maximus (GMAX), medius (GMED) and minimus (GMIN) muscles. At T2, significant changes were measured. The TV and MV of the iliopsoas muscle, gluteus maximus and gluteus medius muscles were decreased, whereas an increase in IMAT in the gluteus medius and minimus muscles were detected (Table 2).

**Figure 2:**
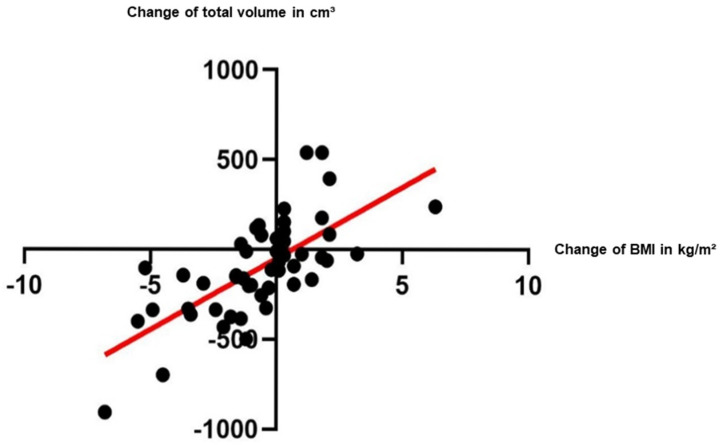
Changes in total volume correlated significantly with changes in Body-Mass-Index (BMI), as calculated by Spearman correlation (r = 0.7, *p* < 0.0001).

Patients‘ Body-Mass-Index (BMI) was calculated at T1 and T2. During the course of treatment, 28 patients lost weight (T1: mean 27.6 kg/m^2^ (±6.2), T2: mean 25.4 kg/m^2^ (±5.8)) and 20 patients gained weight (T1: mean 24.9 kg/m^2^ (±3.8), T2: mean 26.3 kg/m^2^ (±3.8)). The changes in TV correlated significantly with the changes in BMI (Figure 2), as calculated by Spearman correlation, respectively (r = 0.7, *p* < 0.0001).

**Figure 3:**
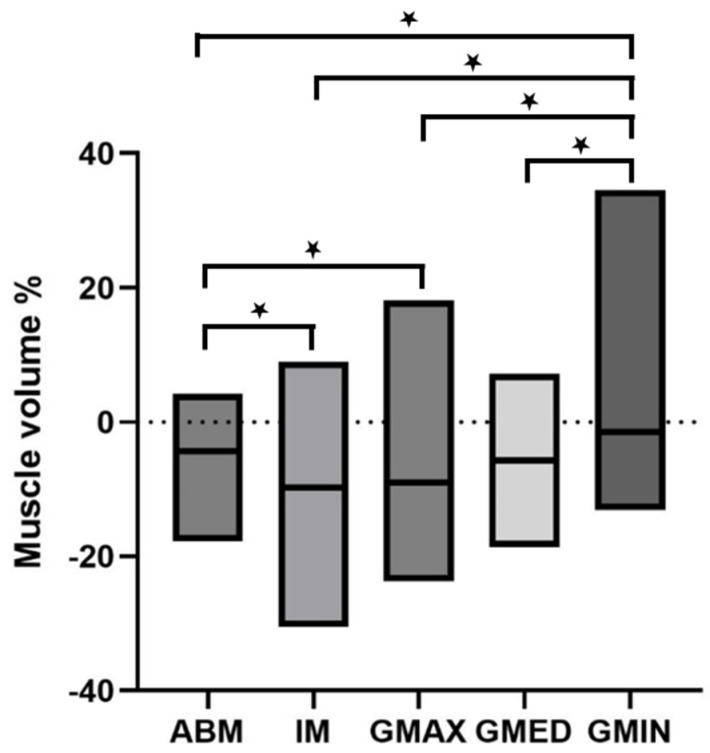
Loss of muscle volume in patients with a decrease in BMI. There was a significant loss of volume detectable in all muscles, except for the M. gluteus minimus. Significant differences of loss of volume between different muscles was calculated by Mann–Whitney test (highlighted by asterisk) and significance level was determined as *p* < 0.05.

Patients with a decrease in BMI lost muscle volume at T2 (Table 3). All muscles were significantly affected except for the M. gluteus minimus. Loss of muscle volume was highest in the iliopsoas muscle (median −9.8%, IQR −4.9-(−14.6%)), followed by M. gluteus max. (median −9.1%, IQR −4.2-(−13.2%)), M. gluteus med. (median −5.8%, IQR −2.9-(−8.9%)), autochthonous back muscles (median −4.3%, IQR −0.75-(−8.0%)) and M. gluteus min. (median −1.5%, IQR +1.5-(−4.6%)) (Figure 3).

**Figure 4:**
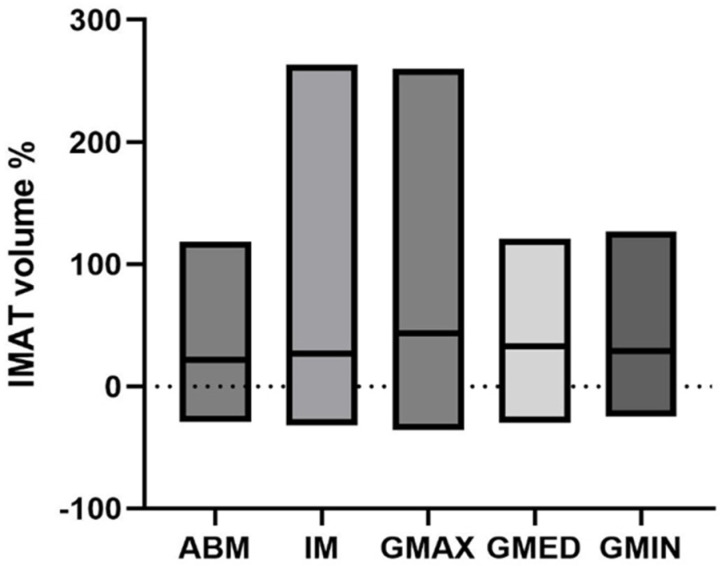
Changes of IMAT in patients with an increase in BMI. There was a significant increase detectable in all muscles between T1 and T2. Increase in IMAT was similar in all muscles. (Mann–Whitney test).

Patients with an increase in BMI showed an increase in intramuscular adipose tissue at T2 (Table 3), which was observed in all muscles. There were no significant differences in IMAT increase between the selected muscles: iliopsoas muscle (median +27.0%, IQR 1.4–62.5%), M. gluteus max. (median +43.6%, IQR 20.0–91.2%), M. gluteus med. (median +32.8%, IQR 12.3–70.6%), autochthonous back muscles (median +22.0%, IQR 4.6–46.0%) and M. gluteus min. (median +29.2%, IQR 10.2–51.8%) (Figure 4).

There were no differences in TV, MV and IMAT volumes detectable between patients with single and tandem autologous stem cell transplantation. Furthermore, the ISS score, which indicates the initial tumor load, was also not predictive of muscle changes in MM patients during treatment.

## 4. Discussion

Loss of muscle volume and strength occurs with advanced age but might also be aggravated by various chronic diseases associated with an underlying systemic inflammatory response, such as cancer [2,23]. In patients suffering from malignant diseases, inflammation induces a pro-catabolic state which leads to elevated protein degradation and hence loss of skeletal muscle tissue. Furthermore, oncologic treatment regimens might additionally affect muscle volume; the use of glucocorticoids in particular has been associated with loss of muscle mass [24,25].

Since multiple myeloma often causes severe bone damage and unstable bone lesions, patients are frequently immobilized over long periods of time or suffer from fear of movement and injury (kinesiophobia), which in turn aggravate loss of muscle mass and functional decline, resulting in an increased risk of falls. Tagliafico et al. examined CT-scans of 74 transplant-eligible MM patients and were able to show that patients with a low skeletal muscle index also suffered from an increased level of bone damage as evaluated by the myeloma spine and bone damage score [26]. Therefore, it is of particular importance to maintain muscle strength in MM patients in order to avoid further injury to an already severely affected bone structure.

One important therapeutic approach to counteract sarcopenia is to include physical exercise, resistance training in particular, in oncologic treatment regimens.

Physical exercise has been shown to have numerous positive effects on cancer patients, such as improved physical function and muscle strength [27,28,29]. However, due to severe bone damage, it is often difficult to perform muscle function testing and, in particular, maximal strength measurements in MM patients. We were therefore interested in evaluating a different approach to assessing muscle changes and training effects in newly diagnosed multiple myeloma patients. Since the majority of patients receive whole-body CT-scans at initial diagnosis and after autologous stem cell transplantation, it seems feasible to use imaging to acquire in-depth information on muscle changes during the course of treatment in MM patients.

So far, studies on sarcopenia in cancer patients have been predominantly performed at one timepoint in order to assess the possible impact on clinical outcomes. For MM patients, the literature on the significance of sarcopenia is still inconsistent. Williams et al. [8] and Abdallah et al. [9] were able to show that sarcopenia is prevalent in MM patients, but no association with overall survival could be determined. Contrary to these results, Nandakumar et al. used a deep learning-based segmentation approach and demonstrated a significant impact of sarcopenia at initial diagnosis on overall survival [30].

We used an artificial intelligence-based three-dimensional segmentation process to evaluate the TV, MV and IMAT of MM patients not only at one timepoint, but during the treatment course.

Our data show that changes in BMI correlated significantly with changes in TV. Patients with a decrease in BMI lost significant muscle volume in all of the examined muscles, except for the GMIN. Loss of muscle volume was more prominent in muscles with more type 2 muscle fibers (fast-twitch, high-energy) whereas muscles with predominantly type 1 fibers (slow-twitch, postural control) were less affected. Patients with an increase in BMI acquired IMAT, which was equally detectable in all of the examined muscles. These changes in skeletal muscle tissue might have significant impact on muscle function. It has been reported that sarcopenia occurs during the aging process and after the age of 50, 1–2% of muscle volume is lost annually. After the age of 60, loss of muscle mass increases to up to 3% per year [31,32]. The patients included in this study were on average 58 years old and we were able to detect median loss of muscle volume of 9.8% in the iliopsoas muscle and 9.1% in the gluteus maximus muscle over an average of 11.8 months, which is significantly more than the age-related average loss of muscle volume, and might thus impact physical function severely. Loss of more type 2 muscle fibers is characteristic for age-related sarcopenia and has been described in the literature [31,33]. Type 2 muscle fibers are crucial for any activity requiring quick powerful movements, for example squatting, sprinting or walking up the stairs. Furthermore, an increase in IMAT in particular has been described as an underlying cause of muscle dysfunction [34,35,36] and fatty degeneration of the gluteus medius and minimus muscles has been associated with fall-related hip fractures in older patients [37,38]. Since we evaluated whole-body CT-scans retrospectively, it is difficult to compare the detected muscle changes on imaging with muscle function and to evaluate whether the loss of muscle volume or the increase in IMAT has a more negative effect on muscle function. Therefore, we are currently using the artificial intelligence-based three-dimensional segmentation method in a prospective clinical trial on an exercise intervention in newly diagnosed MM patients, which should offer further information on muscle function and training effects.

Typically, a single cross-sectional computed tomography image at the third lumbar vertebra is used to estimate total body muscle volume. However, we were able to demonstrate that loss of muscle volume differed between muscles and was more pronounced in high-energy muscles. The artificial intelligence-based three-dimensional segmentation method therefore offers more detailed information on muscle changes during the treatment course, which could aid in tailoring exercise plans more precisely to individual muscle changes with the aim of preventing sarcopenia.

### Limitations

We refrained from computing the skeletal muscle index for individual patients, a customary practice typically executed via a two-dimensional methodology. Furthermore, we did not explore various deep learning algorithms tailored for the segmentation of distinct muscle groups. Additionally, we did not assess the volumes of visceral or subcutaneous adipose tissue, which are essential for a comprehensive evaluation of physical deterioration.

We also acknowledge that there is a significant gender imbalance, which might limit the generalizability of our findings to female MM patients, who may exhibit different patterns of body composition changes. Due to the lack of data on muscle function, clinical implications of the detected muscle changes cannot be assessed. Combining imaging and clinical data on muscle changes during myeloma treatment should therefore be a key direction for further research.

## 5. Conclusions

In conclusion, we were able to demonstrate that the artificial intelligence-based three-dimensional segmentation process is a reliable and time-saving method to acquire in-depth information on sarcopenia in MM patients.

Changes in total volume, muscle volume and intramuscular adipose tissue volume during the course of treatment were detected, which correlated with changes in BMI. Loss of muscle volume was highest in the iliopsoas and the gluteus maximus muscles, whereas increase in IMAT volume was detected in all of the examined muscles.

## Figures and Tables

**Table 1:** Clinical data of patients diagnosed with MM who underwent both initial (T1) and follow-up (T2) whole-body low-dose computed tomography acquisition. VGPR (very good partial response), PR (partial response), MR (minimal response), SD (stable disease).

Patients’ Clinical Data	*n* = 51
**Age (years) at initial diagnosis**	Median 58
	IQR 52–66
	Range: 26–72
**Gender**	
Male	38
Female	13
**Time until follow-up CT-scan (months)**	Mean 11.8 (SD ± 3)
**International Staging System (ISS)**	
1	22
2	8
3	19
Not available	2
**High-dose chemotherapy/autologous stem cell transplantation**	
Single	24
Tandem	27
**Response assessment at T2**	
≥VGPR	44
PR, MR, SD	5
Not available	2
**Body-Mass-Index (BMI) kg/m^2^**	
Weight loss	*n* = 28
T1	27.6 (SD ± 6.2)
T2	25.4 (SD ± 5.8)
Weight gain	*n* = 20
T1	24.9 (SD ± 3.8)
T2	26.3 (SD ± 3.8)
No weight change	*n* = 3

**Table 2:** Depicted are total volume, muscle volume and intramuscular adipose tissue volume (IMAT) of the autochthonous back muscles (ABM), iliopsoas muscle (IM), gluteus maximus muscle (GMAX), gluteus medius muscle (GMED) and gluteus minimus muscle (GMIN) at initial diagnosis (T1) and at follow-up (T2). Differences between the two timepoints were calculated by Wilcoxon matched-pairs signed-rank test and significance level was determined as *p* < 0.05. Median values are shown, as well as the interquartile range (IQR). Significant changes between T1 and T2 are highlighted in bold and by *.

	ABM		IM		GMAX		GMED		GMIN	
	Left	Right	Left	Right	Left	Right	Left	Right	Left	Right
**Total volume in cm^3^**										
**T1**	525 (473–599)	539 (448–604)	314 * (264–364) (*p* = 0.001)	303 * (250–373) (*p* = 0.0004)	601 * (479–717) (*p* = 0.022)	640 * (503–754) (*p* = 0.002)	265 * (237–303) (*p* = 0.02)	267 * (225–315) (*p* = 0.005)	64 (56–73)	70 (60–80)
**T2**	521 (453–597)	526 (436–601)	**304** **(255–357)**	**281** **(245–344)**	**576** **(515–665)**	**600** **(513–718)**	**257** **(227–297)**	**261** **(229–304)**	65 (55–75)	71 (61–82)
**Muscle volume in cm^3^**										
**T1**	481 (409–561)	478 (400–559)	290 * (243–336) (*p* = 0.003)	282 * (233–359) (*p* = 0.0004)	568 * (463–675) (*p* = 0.008)	609 * (486–718) (*p* = 0.0004)	253 * (219–290) (*p* = 0.003)	258 * (218–298) (*p* = 0.001)	61 (52–69)	66 (55–75)
**T2**	483 (402–536)	468 (401–549)	277 (236–333)	260 (223–327)	546 (465–636)	559 (457–686)	240 (213–282)	246 (220–290)	61 (46–68)	66 (55–78)
**IMAT in cm^3^**										
**T1**	38 (28–57)	41 (28–58)	6 (4–12)	7 (5–12)	19 (9–43)	18 * (9–41) (*p* = 0.018)	9 * (6–19) (*p* = 0.03)	8 * (5–18) (*p* = 0.037)	2 * (1–4) (*p* = 0.001)	3 * (1–5) (*p* = 0.018)
**T2**	42 (30–60)	46 (30–62)	7 (5–13)	8 (5–14)	22 (12–43)	**23** **(12–41)**	**11** **(8–16)**	**9** **(6–15)**	**3** **(2–5)**	**3** **(2–5)**

**Table 3:** Changes of total volume, muscle volume and IMAT volume (all muscle groups combined) differed significantly between patients who lost weight (−BMI) and those who gained weight (+BMI). Differences between the two timepoints were calculated by Wilcoxon matched-pairs signed-rank test and significance level was determined as *p* < 0.05. Median values (in cm^3^) are shown, as well as the interquartile range (IQR). Significant changes between T1 and T2 are highlighted in bold and by *.

	*n* = 51	−BMI (*n* = 28)	+BMI (*n* = 20)
**Total volume in cm^3^**			
T1	3653 * (3037–4066) (*p* = 0.005)	3742 * (3393–4213) (*p* < 0.0001)	3340 (2863–4034)
T2	**3461** **(3026–3987)**	**3513** **(3192–3955)**	3433 (2926–4128)
**Muscle volume in cm^3^**			
T1	3329 * (2847–3931) (*p* = 0.0004)	3419 * (3176–4000) (*p* < 0.0001)	3067 (2730–3858)
T2	**3231** **(2809–3664)**	**3226** **(3014–3662)**	3257 (2737–3845)
**IMAT in cm^3^**			
T1	161 * (99–254) (*p* = 0.021)	191 (113.8–396.3)	122 * (96.8–202.8) (*p* = 0.0002)
T2	**179** **(117–267)**	184.5 (117.8–395.8)	**145.5** **(115–248.8)**

## Data Availability

The datasets used and/or analyzed during the current study are available from the corresponding author on reasonable request.

## Abbreviations

AI	Artificial intelligence
BCA	Body composition analysis
BMI	Body-Mass-Index
BOA	Body and organ analysis
CT	Computed tomography
CTDI_vol_	Volumetric computed tomography dose index
HU	Hounsfield Unit
IMAT	Intramuscular adipose tissue volume
IQR	Interquartile range
MM	Multiple myeloma
MV	Muscle volume
nnU-Net	No New U-Net
SD	Standard deviation
T1	Initial examination
T2	Follow-up examination
TV	Total volume
